# Seed Oil of *Lycium barbarum* L. from Qaidam Basin Prevents and Treats UV-Induced Photodamage in BABL/c Mice Skin by Modulating Skin Microbiome and Amino Acid Metabolism

**DOI:** 10.3390/ijms27020731

**Published:** 2026-01-11

**Authors:** Le Han, Yongjing Yang, Benyin Zhang, Yuting Wang, Yiming Ji, Shasha Du, Yongqiang Zou

**Affiliations:** 1 College of Eco-Environmental Engineering, Qinghai University, Xining 810016, China; ys230713000141@qhu.edu.cn (L.H.);; 2 College of Pharmacy, Qinghai University, Xining 810016, China; 3 Qinghai Institute for Endemic Disease Prevention and Control, Xining 810016, China

**Keywords:** seed oil of *Lycium barbarum* L. from Qaidam basin, ultraviolet skin photodamage, oxidative stress, inflammation, 16S rDNA sequencing, metabolomics

## Abstract

Ultraviolet (UV) radiation is a primary environmental factor responsible for skin photodamage, and exposure to UV rays is strongly linked to a variety of skin diseases. This study examined the prophylactic and therapeutic effects of Seed Oil of *Lycium barbarum* L. from the Qaidam basin (QLBSO) in a UV-induced skin photodamage model in BALB/c mice, exploring potential mechanisms by analyzing the skin microbiota and metabolites using 16S rDNA sequencing and metabolomics. The results showed that QLBSO effectively alleviated UV-induced histopathological changes in mouse skin. It also significantly increased the activity of superoxide dismutase (SOD) and catalase (CAT) in UV-damaged skin tissue, while reducing levels of inflammatory cytokines, including interleukin-6 (IL-6), tumor necrosis factor-α (TNF-α), and interleukin-1β (IL-1β), as well as matrix metalloproteinases-1 (MMP-1) and MMP-3. Omics analysis revealed that QLBSO successfully restored the balance of the skin microbiota and corrected disruptions in amino acid metabolism caused by UV exposure. Notably, *Firmicutes_A* and *Kineothrix*, along with cysteine, cystine, glycine, arginine, proline, and choline, were identified as key microbial species and metabolites responsive to QLBSO’s prophylactic and therapeutic effects. In conclusion, QLBSO likely protects against UV-induced skin photodamage by modulating the skin microbiota and amino acid metabolism, providing a scientific foundation for its potential use in skin health protection.

## 1. Introduction

The skin is the body’s largest organ and serves as the primary barrier against external threats. It protects the body from pathogens, physical and chemical injuries, and participates in critical biological functions such as thermoregulation, maintaining water and electrolyte balance, and supporting immune responses. By performing these vital roles, the skin is essential for maintaining overall homeostasis within the body [1]. Beyond genetic factors, ultraviolet (UV, Detailed abbreviations for the entire article are provided in Table S2) radiation from the sun is a major environmental contributor to skin damage and disease. The World Health Organization has classified UV radiation as a “known carcinogen” [2]. Prolonged or excessive exposure to UV radiation can cause photodamage, disrupt immune function, and impair the skin’s physical barrier, leading to a range of skin issues. Research has shown that many skin diseases are closely linked to UV exposure. In addition to various types of skin cancer, such as melanoma, basal cell carcinoma, and squamous cell carcinoma, conditions like chronic photodermatitis, photodermatitis prurigo, solar urticaria, and porphyria cutanea tarda are often triggered or worsened by UV radiation [3,4]. Global statistics indicate that the prevalence of skin diseases has increased by 46.8% since 1990, making them the fourth most common disease category in the Global Burden of Disease (GBD) assessment [5]. Given this rising trend, strategies to mitigate UV-induced skin damage are crucial for preventing the onset of these skin diseases.

The skin is a complex, multilayered organ comprising the epidermis, dermis, and subcutaneous tissue. The epidermis functions as the skin’s primary physical barrier, while the dermis provides elasticity and mechanical strength. The subcutaneous tissue stores energy, cushions external impacts, regulates body temperature, and participates in various metabolic processes [1]. Medium-wave UVB radiation (290–320 nm) is highly energetic but has limited penetrating power, primarily affecting the epidermis. In contrast, long-wave UVA radiation (320–400 nm) has lower intensity but can penetrate deeper into the skin, causing damage to the dermis and subcutaneous tissue [6]. UV radiation generates a substantial amount of reactive oxygen species (ROS), which oxidize lipids, proteins, and DNA in skin cells. The excessive accumulation of ROS and peroxides disrupts the skin’s antioxidant defense mechanisms, exacerbating oxidative stress and triggering multiple intracellular signaling pathways. These processes induce apoptosis in skin cells and compromise the skin’s physical barrier function [7,8]. Concurrently, increased oxidative damage promotes the release of various inflammatory mediators, which activate matrix metalloproteinases (MMPs) that degrade essential skin components, including collagen and elastic fibers. This degradation diminishes the skin’s mechanical strength [9]. Therefore, mitigating oxidative stress and controlling inflammatory responses are critical for reducing UV-induced skin damage.

The skin microbiota is often referred to as the body’s “second barrier” due to its essential role in regulating the skin’s immune system and maintaining the skin’s physical barrier through various mechanisms. Maintaining the balance of the skin microbiota is critical for overall skin health [10]. Dysbiosis, or microbial imbalance, is strongly associated with the onset and progression of many skin diseases, particularly inflammatory conditions such as atopic dermatitis, psoriasis, rosacea, and seborrheic dermatitis [11]. UV-induced skin damage, primarily driven by oxidative stress and inflammation, is also linked to reduced microbiota diversity and disruption of microbial balance. Studies show that UVB radiation decreases the abundance of beneficial resident bacteria, such as *Staphylococcus* and *Streptococcus*, while promoting the overgrowth of opportunistic pathogens like *Staphylococcus aureus* and *Propionibacterium acnes* [12]. In regions with high-altitude UV exposure, where UV radiation is more intense, individuals exhibit reduced α-diversity and increased β-diversity in their skin microbiota. This shift is accompanied by a decrease in beneficial bacteria and an increase in radiation-resistant species, further supporting the link between UV exposure and skin microbiota dysbiosis [13]. This dysbiosis not only results from UV-induced skin damage but also exacerbates the skin’s compromised barrier function, thereby heightening the risk of skin infections. Thus, restoring microbial homeostasis is essential for breaking this damaging cycle and promoting the repair of UV-damaged skin.

The skin microbiota and skin cells collaborate in various metabolic processes, producing a range of metabolites that are essential for maintaining the skin’s barrier function, regulating the microbiota, and modulating skin immunity [14]. Disruptions in these metabolic processes are closely linked to skin damage. Lipids, for example, are crucial for maintaining the integrity of the skin’s stratum corneum and act as key regulators of immune responses in the skin [15]. UV-induced alterations in lipid metabolism disrupt lipid structure, increase epidermal permeability, and exacerbate conditions such as hyperpigmentation and inflammation [16]. Amino acids, fundamental for synthesizing skin structural proteins, play an indispensable role in regulating skin function. UV-induced disturbances in amino acid metabolism compromise the skin’s matrix structure, reducing its mechanical strength [17]. Vitamins, with their antioxidant properties, help repair the skin barrier and mitigate photoaging. However, UV exposure accelerates the degradation of vitamins, triggering skin inflammation and further damage [18]. Consequently, regulating skin metabolism is a critical strategy for alleviating UV-induced skin photodamage.

*Lycium barbarum* L. (*L. barbarum*) is a medicinal and edible berry with highly nutritious properties. China is the world’s largest producer of *L. barbarum* berries, which are widely cultivated in Qinghai, Gansu, and Ningxia provinces of China [19]. These berries are rich in polyphenols, polysaccharides, carotenoids, and other active compounds, and possess numerous health benefits, including immune modulation, anti-aging effects, antioxidant properties, blood pressure reduction, and blood sugar control [20]. *L. barbarum* seed oil, a promising natural oil extracted from *L. barbarum* berry seeds, which is rich in unsaturated fatty acids such as alpha-linolenic acid and oleic acid, as well as micronutrients including vitamin E and Zeaxanthin. It exhibits diverse pharmacological properties, including antioxidant, anti-inflammatory, hypolipidemic, hypoglycemic, and immunomodulatory effects [21]. Recent studies have primarily focused on its extraction methods, component analysis, and bioactivity evaluation [22]. However, the potential protective effects of *L. barbarum* seed oil against UV-induced skin photodamage, particularly regarding its influence on the skin microbiome and metabolism, have not yet been explored. The Qaidam Basin in Qinghai is the second-largest production area of *L. barbarum* in China [23]. Compared to extracts including polysaccharides and fruit oil, the Seed Oil of *L. barbarum* from Qaidam basin (QLBSO) contains elevated levels of alpha-linolenic acid and vitamin E [24]. In addition to fatty acids, such as γ-linolenic acid, palmitoleic acid, linoleic acid, oleic acid, stearic acid, and eicosenoic acid, QLBSO also contains α-linolenic acid and soft fatty acid, which are absent in *L. barbarum* seed oil from Ningxia [25]. In our previous studies, we also detected bioactive components in QLBSO. The contents of unsaturated fatty acids linoleic acid and oleic acid were 67.4% and 19.2%, respectively, while the levels of unsaturated fatty acids stearic acid and palmitic acid were 2.8% and 5.8% respectively. Moreover, the levels of β-carotene, zeaxanthin, and vitamin E were 0.087 mg/100 g, 680 μg/100 g, and 27.4 mg/100 g, respectively [26]. Our preliminary research has shown that Seed Oil of *L. barbarum* cultivated in Qaidam basin (QLBSO) alleviates UVB-induced photodamage in human immortalized keratinocytes (HaCaT) by regulating apoptosis and reducing inflammation. This initial evidence confirms its potential in protecting the skin from UV-induced damage. This study further investigates the prophylactic and therapeutic effects of QLBSO on UV-induced skin damage in a UV-induced BALB/c mouse model. Using high-throughput 16S rDNA sequencing and metabolomics, we identified key species within the skin microbiota and crucial skin metabolites, shedding light on the potential mechanisms of QLBSO via the “skin microbiota metabolite” axis. The goal of this study is to provide a theoretical foundation for exploring the functional properties of QLBSO and expanding its application potential. Additionally, it aims to promote the comprehensive utilization of *L*. *barbarum* berries from Qaidam resources and support the advancement of industrial applications in this area.

## 2. Results

### 2.1. Gas Chromatography-Mass Spectrometry (GC/MS) Component Analysis of QLBSO

**Figure 1:**
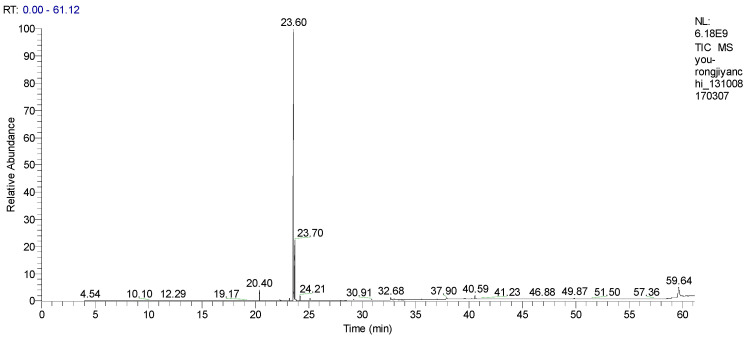
GC/MS Total Ion Chromatogram of Seed Oil of *Lycium barbarum* L. from Qaidam basin.

As shown in Figure 1, separation and identification indicate that QLBSO contains a high level of alkenes, acids, esters, and aromatic hydrocarbons. Biologically active substances, such as Octadecanoic acid (2.33%) and carotene (0.45%), were also detected.

### 2.2. Analysis of Acute Toxicity Test Results for QLBSO

No animal deaths or obvious toxic symptoms were found during the observation period. The mice given QLBSO orally and locally on the skin showed normal behavior, with no significant changes in skin or body weight. The cardiac structures of BALB/c mice remained intact with no myocardial damage or hemorrhage. The mouse’s liver surfaces appeared smooth and non-swollen, the spleen was normal, and the kidneys showed no significant pathological changes. Therefore, we conclude that QLBSO has almost no toxicity.

### 2.3. Histopathological Changes in Skin Tissue

**Figure 2:**
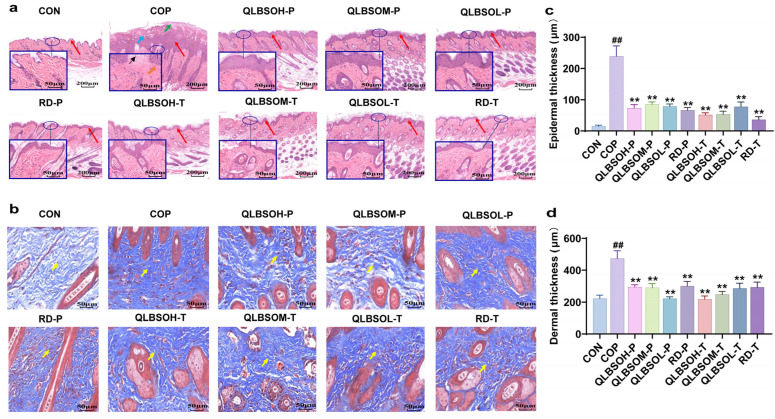
Histopathological analyses of mouse dorsal skin tissue following different QLBSO treatments. (**a**) Mouse skin H&E staining: epidermal thickening (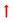), hyperkeratosis (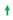), edema (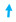), hemorrhage (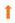), and inflammatory cell infiltration (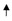). 100× and 400× magnification (scale bar = 200 and 50 µm). (**b**) Mouse skin H&E staining: fibrous tissue proliferation (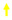). 400× magnification (scale bar = 50 µm); (**c**) Mouse epidermal layer thickness; (**d**) Mouse dermal layer thickness. CON, control group; COP, control positive group; QLBSOH-P: the prophylactic group topically administered 200 μL of QLBSO. QLBSOM-P: the prophylactic group topically administered 100 μL of QLBSO. QLBSOL-P: the prophylactic group topically administered 50 μL of QLBSO. RD-P: the prophylactic reference drug group topically administered 100 μL of VE. QLBSOH-T: the treatment groups topically administered 200 μL of QLBSO. QLBSOM-T: the treatment groups topically administered 100 μL of QLBSO. QLBSOL-T: the treatment groups topically administered 50 μL of QLBSO. RD-T: The treatment reference drug groups topically administered 100 μL of VE. Values are presented as mean ± SD (*n* = 3). Statistical analysis was performed using one-way ANOVA followed by Tukey’s post hoc test for multiple comparisons. ^##^ indicate comparisons with the CON group (*p* ≤ 0.01); ** indicate comparisons with the COP group (*p* ≤ 0.01).

H&E staining (Figure 2a) revealed that skin from CON (control group) mice displayed well-organized epidermal, dermal, and subcutaneous structures, without fibrous tissue proliferation or inflammatory cell infiltration. In contrast, COP (control positive group) mice exhibited clear pathological alterations, including epidermal thickening, partial parakeratosis of the stratum corneum, moderate dermal edema, inflammatory cell infiltration, and local hemorrhage. As shown in Figure 2c,d, both epidermal and dermal thickness were significantly greater in COP mice than in CON mice (*p* ≤ 0.01), indicating that UV irradiation induced hyperkeratosis and thickening of the superficial skin layers. Compared with the COP group, topical treatment with QLBSO or VE (Vitamin E) markedly reduced epidermal thickness and inflammatory cell infiltration, demonstrating substantial histological improvement. Epidermal and dermal thickness were both significantly lower after QLBSO and VE application than in COP mice (*p* ≤ 0.01). Among these treatments, QLBSOH-P (200 μL QLBSO prophylactic group) and QLBSOH-T (200 μL QLBSO treatment group) produced the most pronounced reduction in epidermal thickness. In comparison, QLBSOL-P (50 μL QLBSO prophylactic group) and QLBSOH-T achieved the strongest decrease in dermal thickness. Although epidermal thickness in QLBSO-treated mice remained slightly higher than in the VE group, dermal thickness was marginally lower. Overall, therapeutic topical administration was superior to prophylactic topical administration.

Masson staining was performed to evaluate alterations in dermal fibrous tissue [27]. As shown in Figure 2b, skin from CON mice displayed well-organized collagen fibers with no evidence of hyperplasia. In contrast, COP mice exhibited pronounced fibrous tissue proliferation, indicating extensive UV-induced dermal remodeling. Topical application of QLBSO and VE markedly reduced fibrous tissue hyperplasia compared with COP mice. In the prophylactic dosing groups, QLBSOL-P mice showed higher levels of fibrous tissue proliferation than RD-P (100 μL VE prophylactic reference drug group) mice. Under the therapeutic strategy, QLBSOL-T (50 μL QLBSO treatment group) treatment produced better outcomes than RD-P. Among all groups, QLBSOM-P (100 μL QLBSO prophylactic group) and QLBSOM-T (100 μL QLBSO treatment group) achieved the most pronounced improvement, with collagen organization closely resembling that of the CON group. No statistically significant differences were detected between the prophylactic and therapeutic dosing strategies.

Histopathological examination confirmed that UV irradiation caused substantial skin thickening and fibrous tissue proliferation, validating the successful establishment of a mouse skin photodamage model [28]. Both prophylactic and therapeutic applications of QLBSO effectively alleviated these UV-induced structural abnormalities. These results indicate that QLBSO significantly mitigates UV-induced damage in mouse skin tissue.

### 2.4. Regulation of QLBSO on Oxidative Stress, Inflammatory Markers, and Matrix Metalloproteinases in UV-Damaged BALB/c Mouse Skin

**Figure 3:**
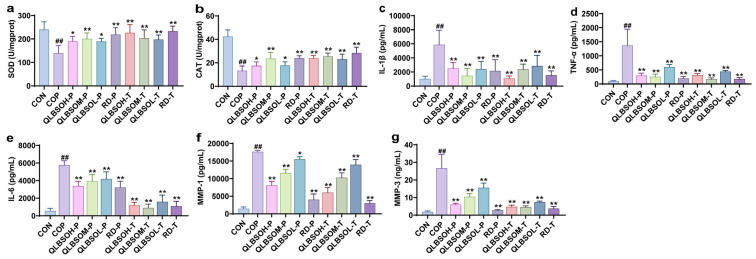
Effects of QLBSO on Oxidative Stress, Inflammatory Markers, and Matrix Metalloproteinases in UV-Damaged BALB/c Mouse Skin. (**a**,**b**) Effects of Different Concentrations and Durations of QLBSO and VE on the Activity of Antioxidant Enzymes SOD and CAT; (**c**–**e**) Effects of Different Concentrations and Durations of QLBSO and VE on the levels of inflammatory cytokines IL-1β, TNF-α, and IL-6. (**f**,**g**) Effects of Different Concentrations and Durations of QLBSO and VE on the Levels of Inflammatory Cytokines MMP-1 and MMP-3. Mouse dermal layer thickness. CON, control group; COP, control positive group; QLBSOH-P: the prophylactic group topically administered 200 μL of QLBSO. QLBSOM-P: the prophylactic group topically administered 100 μL of QLBSO. QLBSOL-P: the prophylactic group topically administered 50 μL of QLBSO. RD-P: the prophylactic reference drug group topically administered 100 μL of VE. QLBSOH-T: the treatment groups topically administered 200 μL of QLBSO. QLBSOM-T: the treatment groups topically administered 100 μL of QLBSO. QLBSOL-T: the treatment groups topically administered 50 μL of QLBSO. RD-T: the treatment reference drug group topically administered 100 μL of VE. Values are presented as mean ± SD (*n* = 8). Statistical analysis was performed using one-way ANOVA followed by Tukey’s post hoc test for multiple comparisons. ^##^ indicate comparisons with the CON group (*p* ≤ 0.01); * and ** indicate comparisons with the COP group (*p* ≤ 0.05 and *p* ≤ 0.01, respectively).

As shown in Figure 3a,b, SOD and CAT activities in the COP mouse skin were significantly lower than those in the CON group (*p* ≤ 0.01). Compared with COP, topical application of QLBSO and VE markedly increased SOD and CAT activities in QLBSOH-P and QLBSOL-T mice (*p* ≤ 0.05), while other treatment groups showed even greater increases (*p* ≤ 0.01). SOD activity rose in a dose-dependent manner with increasing QLBSO concentration. For CAT, QLBSOL-P and QLBSOM-T showed the strongest effects, while the overall efficacy of QLBSO remained slightly below that of VE. These findings suggest that QLBSO and VE mitigate UV-induced oxidative stress in mouse skin by enhancing antioxidant enzyme activity.

Inflammation is a key feature of UV-induced skin injury, with IL-1β, IL-6, and TNF-α serving as major pro-inflammatory mediators [29]. As shown in Figure 3c–e, IL-1β, IL-6, and TNF-α levels were significantly elevated in COP mice compared with CON (*p* ≤ 0.01). Topical application of QLBSO and VE significantly reduced the levels of these cytokines in mouse skin (*p* ≤ 0.01). In the prophylactic regimen, IL-1β and TNF-α levels in QLBSOM-P mice were closest to those in CON, and IL-6 declined in a dose-dependent manner with increasing QLBSO dosage. In the therapeutic regimen, IL-1β also decreased with higher QLBSO concentration, while IL-6 and TNF-α levels in QLBSOM-T mice approximated those of CON. Notably, IL-1β levels in QLBSOM-P and QLBSOH-T were slightly lower than in RD-P and RD-T (100 μL VE treatment reference drug group), respectively. In contrast, TNF-α and IL-6 levels were somewhat higher in both QLBSO treatment and prevention groups than in the positive controls.

Matrix MMPs accelerate the breakdown of the extracellular matrix, leading to structural alterations in collagen and elastin, and play key roles in tissue remodeling, wound healing, and inflammation. Pro-inflammatory cytokines such as IL-1β, IL-6, and TNF-α can upregulate MMP expression, thereby amplifying UV-induced structural damage to the skin [30]. As shown in Figure 3f,g, MMP-1 and MMP-3 activities in the COP group mouse skin were significantly elevated compared with those in the CON group (*p* ≤ 0.01). Topical application of QLBSO and VE markedly reduced MMP-1 and MMP-3 activities relative to COP (control positive group), with significant reductions observed in all groups except QLBSOL-P, which showed a moderate but significant decrease in MMP-1 (*p* ≤ 0.05). In the QLBSO prophylactic regimen, MMP-1 and MMP-3 levels declined in a dose-dependent manner with increasing QLBSO concentration (*p* ≤ 0.01). Similarly, in the therapeutic regimen, both MMP-1 and MMP-3 levels decreased progressively with higher QLBSO doses. Among the treatment groups, MMP-3 levels were comparable between QLBSOM-T and QLBSOH-T, suggesting a plateau in response at higher concentrations. Notably, MMP-1 and MMP-3 levels in QLBSO-treated mice remained slightly higher than those in the VE positive control group.

These findings indicate that UV irradiation causes photodamage by inducing oxidative stress, stimulating inflammatory pathways, and elevating matrix metalloproteinase expression. Both prophylactic and therapeutic applications of QLBSO effectively mitigated these effects by reducing the activities of SOD, CAT, IL-1β, TNF-α, IL-6, MMP-1, and MMP-3. Collectively, these results demonstrate that QLBSO exerts both prophylactic and reparative effects against UV-induced skin injury, with therapeutic application generally producing stronger protective outcomes than prophylactic treatment.

### 2.5. Effects of QLBSO on UV-Induced Photodamage and Skin Surface Microbiota in Mice

**Figure 4:**
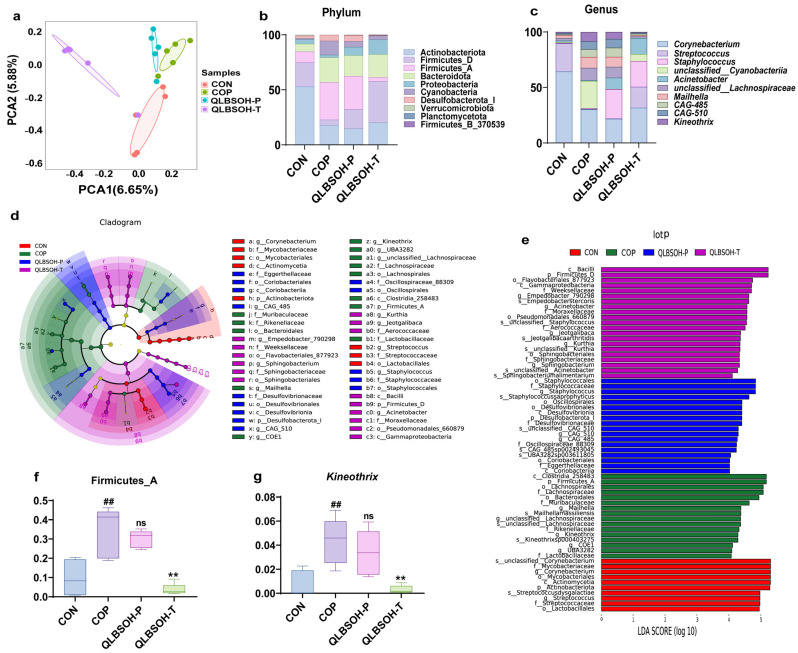
Effects of QLBSO on Skin Microbiota of UV-Damaged Mice. (**a**) Principal component analysis (PCA) of QLBSO on UV-damaged mouse skin microbiota; (**b**,**c**) Representing the relative abundance at the phylum and genus family levels in UV-irradiated BALB/c mouse skin, respectively; (**d**,**e**) LDA-based cladogram and distribution histograms, respectively; (**f**) *Firmicutes_A* box plot; (**g**) *Kineothrix* box plot; CON, control group; COP, control positive group; QLBSOH-P, the prophylactic group topically administered 200 μL of QLBSO. QLBSOH-T: the treatment groups topically administered 200 μL of QLBSO. Values are presented as mean ± SD (*n* = 5). Statistical analysis was performed using one-way ANOVA followed by Tukey’s post hoc test for multiple comparisons. ^##^ indicate comparisons with the CON group (*p* ≤ 0.01); ** indicate comparisons with the COP group (*p* ≤ 0.01). “ns” indicate comparisons with the COP group (*p* > 0.05).

We investigated the effects of QLBSO on the skin microbiota of UV-induced photodamaged mice using 16S rDNA high-throughput sequencing. Principal Component Analysis (PCA) (Figure 4a) showed clear separation among CON, COP, QLBSOH-P, and QLBSOH-T groups. UV irradiation caused significant disruption of the normal skin microbiota, while the microbial composition in QLBSOH-P and QLBSOH-T mice more closely resembled that of CON mice. Gene sequencing identified 22 bacterial phyla and 507 genera across all samples. Accumulation plots were generated at both the phylum and genus levels, displaying the top 10 most abundant taxa. As shown in Figure 4b, at the phylum level, the relative abundances of *Firmicutes_A* and *Bacteroidetes* differed markedly between the CON and COP groups. At the genus level (Figure 4c), COP group mice exhibited notable changes in *Streptococcus*, *Staphylococcus*, and *Kineothrix* abundance. Following QLBSO treatment, the relative abundance of these genera showed a clear downward trend, suggesting partial restoration of microbial balance.

We conducted LEfSe (LDA Effect Size) analysis to identify microbial biomarkers showing significant differences in skin surface abundance among mouse groups, using thresholds of LDA ≥ 4 and *p* ≤ 0.05. As shown in Figure 4d,e, at the phylum level, *Actinobacteriota* dominated the skin microbiota of CON mice, whereas *Firmicutes_A* was enriched in COP and QLBSOH-T mice. QLBSOH-P mice showed enrichment in *Desulfobacterota_I*. At the genus level, *Corynebacterium* and *Streptococcus* were enriched in CON mice, while COP mice exhibited higher levels of *unclassified__Lachnospiraceae*, *Kineothrix*, *Mailhella*, *UBA3282*, and *COE1*. QLBSOH-P mice were enriched in *CAG-485*, *Staphylococcus*, and *CAG-510*, whereas QLBSOH-T mice showed enrichment in *Kurthia*, *Acinetobacter*, *Sphingobacterium*, *Empedobacter_790298*, and *Jeotgalibaca*. These taxa were identified as major biomarkers differentiating the groups. Univariate ANOVA results (Figure 4f,g) showed that *Firmicutes_A* and *Kineothrix* were significantly elevated in COP mice (*p* ≤ 0.01) but markedly decreased following QLBSO intervention. The decrease was greater in QLBSOH-T mice (*p* ≤ 0.01), whose microbiota composition more closely resembled that of CON mice. These findings suggest that *Firmicutes_A* and *Kineothrix* play key roles in the microbial response to QLBSO. Overall, both prophylactic and therapeutic QLBSO treatments promoted recovery of the skin microbiota, with therapeutic intervention producing a more pronounced restorative effect.

### 2.6. Effects of QLBSO on Metabolites in UV-Induced Photodamaged Skin Tissue of Mice

**Figure 5:**
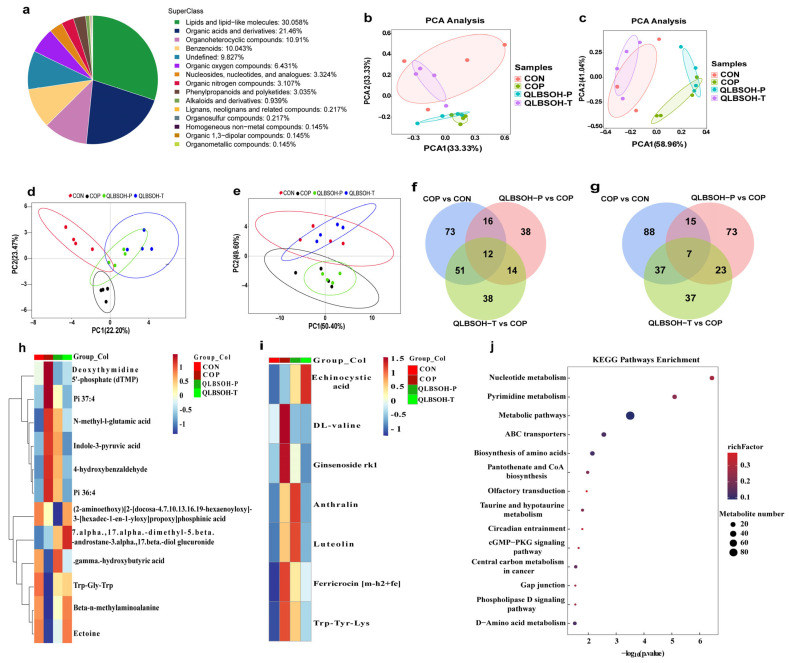
Non-targeted metabolomic analysis of UV-damaged mouse skin treated with QLBSO. (**a**) Metabolite classification in UV-damaged mouse skin; (**b**,**c**) Principal Component Analysis (PCA) in Positive and Negative Ion Modes (PLS-DA); (**d**,**e**) Partial Least Squares Discriminant Analysis in Positive and Negative Ion Modes; (**f**,**g**) Venn Diagram of Differential Metabolites in Positive and Negative Ion Modes; (**h**,**i**) Heatmap of Differential Metabolites in Positive and Negative Ion Modes; (**j**) KEGG enrichment pathway bubble chart. CON, control group; COP, control positive group; QLBSOH-P, the prophylactic group topically administered 200 μL of QLBSO. QLBSOH-T: the treatment groups topically administered 200 μL of QLBSO.

We performed non-targeted metabolomics to evaluate the effects of different QLBSO intervention strategies on skin metabolites in mice. In total, we identified 1386 metabolites, including 807 detected in positive ion mode and 579 in negative ion mode. Metabolite classifications are presented in Figure 5a, with the top three categories being lipids and lipid-like molecules (30.058%), organic acids and derivatives (21.46%), and Organoheterocyclic compounds (10.91%). Principal component analysis (PCA) and partial least squares–discriminant analysis (PLS-DA) score plots (Figure 5b–e) showed clear separation between CON and COP groups, indicating significant UV-induced metabolic disturbances in mouse skin. Compared with COP mice, QLBSOH-P and QLBSOH-T samples exhibited metabolic profiles more closely resembling those of CON mice, suggesting that topical QLBSO application partially restored normal skin metabolism.

Using a screening threshold of *p* ≤ 0.05, we identified 522 differential metabolites among the CON, COP, QLBSOH-P, and QLBSOH-T groups—280 in positive ion mode and 242 in negative ion mode (Figure 5f,g). Of these, twelve metabolites in positive ion mode and seven in negative ion mode were shared across all four groups. Cluster heatmap analysis of these common differential metabolites (Figure 5h,i) revealed that metabolites such as ectoine, Trp-Gly-Trp, and β-N-methylaminoalanine, which were initially abundant in CON mice, decreased markedly after UV irradiation but increased again following QLBSO intervention. These findings indicate that QLBSO treatment effectively counteracted UV-induced metabolic disruptions in mouse skin.

Metabolites that were initially present at low levels in the CON group, such as anthralin, DL-valine, and N-methyl-L-glutamic acid, showed marked increases following UV irradiation but decreased significantly after topical application of QLBSO. These findings indicate that QLBSO effectively alleviates UV-induced metabolic disturbances in mouse skin and helps restore metabolite levels in photodamaged tissue. Notably, among the differentially expressed metabolites, four cations and three anions were classified as amino acids, peptides, and analogs.

We further performed enrichment analysis of differentially expressed metabolites across the four groups (CON, COP, QLBSOH-P, and QLBSOH-T) using *p* ≤ 0.05 as the significance threshold to identify metabolic pathways modulated by QLBSO under both prophylactic and therapeutic conditions. As shown in Figure 5j, these metabolites were primarily enriched in pathways associated with amino acid, nucleotide, and pyrimidine metabolism. Within the amino acid-related pathways, two major routes were identified: Biosynthesis of amino acids and D-amino acid metabolism. Collectively, these results suggest that QLBSO may exert its prophylactic and therapeutic effects against UV-induced skin photodamage primarily by regulating amino acid metabolism in the skin.

### 2.7. Effects of QLBSO on Amino Acid Metabolism in UV-Induced Photodamaged Skin Tissue of Mice

**Figure 6:**
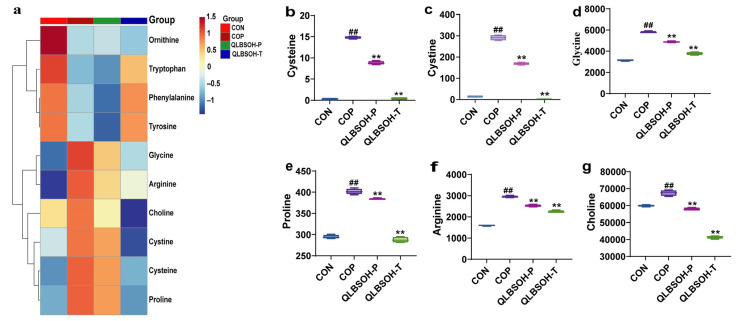
Targeted metabolomic analysis of UV-damaged mouse skin by QLBSO. (**a**) Heatmap of amino acids in UV-damaged mouse skin treated with QLBSO; (**b**–**g**) Boxplot of cysteine, cystine, glycine, proline, arginine, and choline Content. CON, control group; COP, control positive group; QLBSOH-P, the prophylactic group topically administered 200 μL of QLBSO. QLBSOH-T: the treatment groups topically administered 200 μL of QLBSO. Values are presented as mean ± SD (*n* = 4). Statistical analysis was performed using one-way ANOVA followed by Tukey’s post hoc test for multiple comparisons. ^##^ indicate comparisons with the CON group (*p* ≤ 0.01); ** indicate comparisons with the COP group (*p* ≤ 0.01).

We further investigated the effects of QLBSO on amino acid metabolism in UV-damaged mouse skin using targeted amino acid metabolomics. In total, 29 differentially expressed amino acids were identified, and the top 10 ranked by ascending *p*-value were selected for cluster heatmap analysis (Figure 6a). The results showed that amino acids such as ornithine, tryptophan, phenylalanine, and tyrosine, which were present at high levels in the CON group, decreased significantly after UV irradiation. Following both prophylactic and therapeutic QLBSO interventions, their levels increased markedly, approaching those observed in the CON group. Conversely, amino acids and derivatives initially present at low concentrations, such as cysteine, cystine, glycine, proline, arginine, and choline, showed pronounced elevation after UV exposure. However, their levels declined following both QLBSO prevention and treatment, restoring them closer to baseline values. These findings suggest that QLBSO effectively mitigates UV-induced disturbances in amino acid metabolism and promotes metabolic homeostasis in skin tissue. One-way ANOVA of these 10 amino acids and derivatives revealed that cysteine, cystine, glycine, proline, arginine, and choline levels increased significantly (*p* ≤ 0.01) in UV-irradiated skin. After topical QLBSO application, their concentrations decreased dramatically (*p* ≤ 0.01), with the therapeutic regimen (QLBSO-T) producing a stronger effect than the prophylactic regimen (QLBSO-P) (Figure 6b–g). Collectively, these results indicate that QLBSO not only prevents but also more effectively reverses UV-induced metabolic imbalances in amino acid pathways.

### 2.8. Correlation Analyses

**Figure 7:**
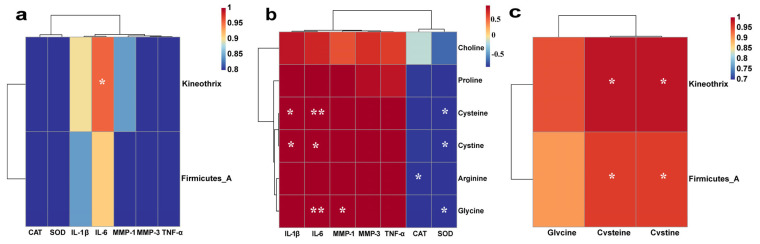
Correlation Analyses. (**a**) Correlation between skin bacterial genera and skin indicators; (**b**) Correlation between amino acids and skin indicators; (**c**) Correlation between amino acids and skin bacterial genera. * and ** indicate comparisons *p* ≤ 0.05 and *p* ≤ 0.01, respectively.

We analyzed the relationships among key differentially abundant bacteria (*Firmicutes_A* and *Kineothrix*), amino acids (cysteine, cystine, glycine, proline, arginine), their derivative choline, oxidative stress markers (SOD, CAT), inflammatory cytokines (IL-1β, IL-6, TNF-α), and matrix metalloproteinases (MMP-1, MMP-3) using Pearson correlation hierarchical clustering. The results showed that *Kineothrix* was significantly positively correlated with IL-6 in skin tissue (*p* ≤ 0.05) (Figure 7a). Cysteine, cystine, and glycine exhibited significant negative correlations with SOD (*p* ≤ 0.05) and significant positive correlations with IL-6 (*p* ≤ 0.05). Notably, cysteine and glycine displayed highly significant positive correlations with IL-6 (*p* ≤ 0.01). Cysteine and cystine were also positively correlated with IL-1β, while glycine correlated positively with MMP-1, and arginine correlated negatively with CAT. Importantly (Figure 7b), *Kineothrix* and the three amino acids—cysteine, cystine, and glycine—all showed strong associations with IL-6 levels. Further analysis revealed that cysteine and cystine were significantly positively correlated with both *Kineothrix* and *Firmicutes_A* (*p* ≤ 0.05), suggesting that these microbial and metabolic changes may jointly influence UV-induced skin inflammation (Figure 7c).

## 3. Discussion

UV irradiation induces oxidative stress and inflammation in the skin, leading to collagen fiber degradation and fragmentation of collagen bundles within the extracellular matrix. This damage compromises both the skin’s mechanical integrity and immune barrier, manifesting as erythema, desquamation, hyperkeratosis, and epidermal necrosis [7]. In this study, H&E and Masson staining demonstrated that topical application of QLBSO effectively alleviated UV-induced epidermal thickening and dermal fibrous tissue proliferation in both prophylactic and therapeutic models (Figure 2). These findings align with previous reports showing that Chuanxiong oil mitigates UV-induced skin thickening and fibrous tissue hyperplasia in BALB/c mice [31]. Endogenous antioxidant enzymes protect the skin from oxidative injury through various mechanisms, including free radical scavenging and metal ion chelation [32]. Under UV-induced oxidative stress, SOD neutralizes ROS, such as the superoxide anion (O_2_^−^), the hydroxyl radical (·OH), and the peroxy radical (ROO·), converting them into less reactive peroxides. CAT then catalyzes the breakdown of these peroxides into water and oxygen, thereby maintaining redox homeostasis and protecting oxidative damage to skin cells and extracellular structures [33]. Our results showed that topical QLBSO application effectively restored the reduced SOD and CAT activities in UV-irradiated mouse skin under both prophylactic and therapeutic conditions (Figure 3a,b). These findings are consistent with previous observations in UV-induced BALB/c mice, where zeaxanthin extracted from *L. barbarum* also reversed SOD and CAT depletion [34].

UV-induced oxidative stress produces large amounts of ROS, which activate signaling pathways such as NF-κB and MAPK, leading to the upregulation of inflammatory mediators, including IL-1β, IL-6, and TNF-α, thereby amplifying inflammation [35]. TNF-α, a major pro-inflammatory cytokine secreted by macrophages, lymphocytes, and other immune cells, enhances the expression of adhesion molecules such as MHC-I, ICAM-1, and VCAM-1, promoting inflammatory cell infiltration [36]. IL-1β is among the earliest cytokines activated after UV exposure; it induces vasodilation, fever, and leukocytosis, stimulates IL-6 production, and activates matrix metalloproteinase (MMPs) expression, contributing to photoaging and extracellular matrix degradation [37]. IL-6, a multifunctional cytokine secreted by monocytes, fibroblasts, and endothelial cells, further amplifies inflammatory signaling and promotes fibroblast-mediated expression of MMP-1 and MMP-9, leading to collagen breakdown and skin photodamage [38]. MMPs are zinc-dependent endopeptidases responsible for degrading components of the extracellular matrix (ECM) [39]. Among them, MMP-1 primarily cleaves type I and type III collagen, while MMP-3 degrades multiple structural matrix proteins. Elevated expression of these enzymes disrupts the skin’s supportive framework and accelerates tissue injury [40]. In this study, topical application of QLBSO effectively reversed the UV-induced elevation of IL-1β, IL-6, TNF-α, MMP-1, and MMP-3 in mouse skin under both prophylactic and therapeutic conditions (Figure 3c–g). These results are consistent with reports that *Clerodendranthus spicatus* suppresses UV-induced increases in IL-1β, IL-6, TNF-α, MMP-1, and MMP-3 in a BALB/c mouse photodamage model [41], and with findings showing that polydopamine nanoparticles downregulate IL-1β, TNF-α, MMP-1, and MMP-3 expression in a similar model [42]. The above findings indicate that QLBSO exerts antioxidant effects by scavenging free radicals and regulating antioxidant enzyme activity, and demonstrates anti-inflammatory effects by inhibiting the release of pro-inflammatory cytokines and interfering with inflammatory signal transduction. These actions are attributable to the numerous bioactive components within QLBSO, which synergistically interact to exert antioxidant and anti-inflammatory effects. Among these, Octadecanoic Acid in QLBSO exerts antioxidant activity by enhancing cellular superoxide dismutase (SOD) and catalase (CAT) activity [43]. Oleic acid effectively neutralizes intracellular ROS production [44]. Additionally, β-carotene neutralizes peroxy radicals, interrupts radical chain reactions, and protects lipid structures, including those in cell membranes, from peroxidation [45]. Vitamin E reacts with lipid peroxy radicals, interrupting the chain reaction of lipid peroxidation, downregulating inflammatory responses, and safeguarding the integrity of cell membranes and lipoproteins [46]. Alpha-linolenic acid can reduce inflammation by inhibiting cyclooxygenase (COX) and 5-lipoxygenase (5-LOX) [47]. Phytosterols regulate immune cell function and inhibit the production of pro-inflammatory cytokines, exerting anti-inflammatory effects [48]. The therapeutic efficacy of QLBSO in this study was notably greater than its prophylactic efficacy. This difference may result from UV irradiation disrupting the skin’s physical barrier, thereby increasing skin permeability [49] and facilitating deeper penetration of QLBSO into the tissue, thereby sustaining its reparative effects. Additionally, studies have shown that UV-induced acceleration of skin blood flow can enhance the distribution efficiency of topical medications [50], potentially contributing to the superior therapeutic performance observed with QLBSO treatment.

The skin functions as a dynamic and complex ecosystem inhabited by trillions of microorganisms that together constitute the skin microbiome [51]. This microbial community is crucial for maintaining skin homeostasis, modulating immune responses, and defending against pathogenic invasion. Numerous studies have demonstrated that disturbance of microbial balance on the skin surface leads to impaired physiological functions and compromised skin health [52]. Under normal conditions, *Actinobacteria*, *Firmicutes*, *Proteobacteria*, and *Bacteroidetes* represent the dominant bacterial phyla of healthy skin. UV irradiation disrupts this balance by decreasing the relative abundance of *Actinobacteria* and *Bacteroidetes* while increasing that of *Firmicutes* [53,54]. In our study, LEfSe analysis revealed that UV irradiation significantly altered the abundance of *Actinobacteriota*, *Firmicutes_A*, and *Desulfobacterota_I* on the mouse skin surface (Figure 4d,e), with *Firmicutes_A* showing a marked elevation in the COP group. Following intervention with both QLBSO treatment methods, *Firmicutes_A* levels declined substantially, particularly in the QLBSH-T group, where a significant reduction was observed (Figure 4f). *Actinobacteria*, a group of Gram-positive bacteria, represent one of the most abundant phyla within a healthy skin microbiome and rely on the skin as a key ecological niche [55].

Members of the genus *Corynebacterium* utilize skin and sweat-derived lipids as nutrient sources and help maintain microbiome stability by producing antimicrobial compounds [56]. *Firmicutes_A*, a subclass of *Firmicutes*, includes genera such as *Streptococcus*, *Kineothrix*, and *Staphylococcus*. Among these, *Streptococcus* is typically regarded as a core and beneficial component of the skin microbiota. In contrast, *Staphylococcus* is strongly linked to inflammatory skin disorders such as atopic dermatitis and can exacerbate cutaneous inflammatory responses [57]. Multiple studies have shown that various common inflammatory skin diseases feature a pronounced decrease in the relative abundance of *Actinobacteria* and a concurrent increase in *Firmicutes*. For example, acne lesions display a marked reduction in *Actinobacteria* and an elevated proportion of *Firmicutes* compared with unaffected skin [58]. Similarly, the skin microbiome of psoriasis patients shows a consistent pattern of decreased *Actinobacteria* and increased *Firmicutes* abundance [59]. *Desulfobacterota_I* comprise sulfur-reducing bacteria capable of promoting inflammation through the production of hydrogen sulfide (H_2_S) and lipopolysaccharides (LPS). Previous research suggests that sulfates produced by these bacteria can damage intestinal epithelial cells and trigger inflammatory responses [60]. In this study, we identified *Firmicutes* and *Kineothrix* (a genus within *Firmicutes*) as key skin surface microbes associated with QLBSO-mediated alleviation of UV-induced skin photodamage in mice (Figure 4f,g). Correlation analysis revealed a significant positive relationship between *Kineothrix* and IL-6 expression. Notably, *Firmicutes* has also been shown to correlate positively with serum IL-6 levels in a type 2 diabetic rat model (Figure 7a) [61]. Therefore, QLBSO may attenuate inflammatory responses in UV-induced skin photodamage by reducing the relative abundance of *Kineothrix* on the skin surface of mice. UV radiation disrupts the skin’s microbiome structure, altering skin homeostasis. Unsaturated fatty acids, including palmitic acid and linoleic acid, can reduce the proportion of *Firmicutes* in the gut microbiota while increasing the level of *Bacteroidetes* [62]. Phytosterols have natural surfactant properties that interfere with the colonization of harmful bacteria (*Propionibacterium acnes*) on the skin surface. Beta-carotene, zeaxanthin, and vitamin E protect the beneficial skin microbiota (*Staphylococcus epidermidis*) and safeguard the skin microbiome [63,64]. These components provide the foundation for QLBSO to regulate microbial communities.

Amino acids play essential roles in skin physiology, including maintaining hydration, promoting collagen synthesis, facilitating tissue repair, and strengthening immune defense. The hydrophilic functional groups in their molecular structures, such as amino (-NH_2_), carboxyl (-COOH), and thiol (-SH) groups, form hydrogen bonds with water molecules, allowing amino acids to attract and retain moisture and thereby sustain optimal skin hydration [65]. When the skin is injured, amino acids stimulate the proliferation and differentiation of skin cells, accelerating tissue repair and regeneration [66]. In addition, several amino acids, including cysteine, exhibit potent antioxidant activity. By scavenging excess free radicals, they minimize oxidative damage to skin cells, preserve structural integrity, and maintain the function of the skin’s physical barrier [67]. Research suggests that UV irradiation disrupts the skin’s antioxidant system, leading to oxidative stress. This intense oxidative environment leads to carbonylation or direct degradation of proteins in the dermis and epidermis, releasing free amino acids into the interstitial spaces and elevating amino acid levels [68]. In this study, metabolomic analysis showed that QLBSO significantly reversed the UV-induced abnormal elevation of five amino acids, cysteine, cystine, glycine, proline, and arginine, as well as their derivative choline in mouse skin (Figure 6). These findings suggest that these amino acids contribute critically to QLBSO’s protective and therapeutic effects against UV-induced skin injury. Cysteine is a sulfur-containing amino acid and a precursor for glutathione (GSH). Reactive oxygen species (ROS) induce structural changes in Keap1, causing Nrf2 accumulation and enhanced intracellular GSH synthesis. This process scavenges ROS and mitigates oxidative damage to the skin. However, excessive cysteine inhibits Keap1’s conformational changes, enhancing Keap1 binding to Nrf2. This blocks downstream antioxidant gene expression, exacerbating oxidative stress in the skin [69]. Cystine, composed of two cysteine molecules linked by a disulfide bond, reinforces keratin fibers and provides additional antioxidant protection [70].

Consistent with these findings, we observed that UV irradiation markedly increased the levels of both cysteine and cystine in mouse skin, a pattern similar to the elevated Cysteine content previously reported in UV-exposed HeLa cells [71]. Further correlation analysis demonstrated that cysteine and cystine exhibited strong positive associations with *Firmicutes* and *Kineothrix* on the mouse skin surface (Figure 7c). In a mouse adenomyosis model, *Firmicutes*, particularly *Lactobacillus*, were shown to synthesize or release cysteine through metabolic enzymes such as cysteine β-synthase and cysteine γ-lyase, thereby increasing cysteine concentrations in the host gut [72]. Studies using U937 monocytes have also shown that cysteine and cystine upregulate IL-1β expression [73], which in turn activates downstream signaling cascades leading to IL-6 production. This observation aligns with our findings that cysteine and cystine levels were significantly and positively correlated with the inflammatory mediators IL-1β and IL-6 (Figure 7b). Glycine, the most abundant amino acid in skin collagen, directly participates in collagen fiber assembly, maintaining dermal structural integrity [65]. Beyond its structural role, Glycine contributes to skin hydration, enhances immune defense, and exhibits anti-inflammatory properties. Notably, in the BV2 microglial cell inflammation model, Glycine markedly promoted IL-6 secretion [74], consistent with our results showing a significant increase in glycine levels in UV-irradiated mouse skin and a strong positive correlation with IL-6 (Figure 7b). Correlation analysis further revealed that glycine, cysteine, and cystine were significantly negatively correlated with SOD levels in mouse skin (Figure 7b). This finding agrees with previous reports showing that mixtures of multiple amino acids, including glycine and cysteine, can markedly suppress SOD expression [75]. HydroxyProline (HYP), a characteristic amino acid unique to collagen, serves as a reliable biochemical marker for assessing collagen content and skin matrix integrity [76]. UV-induced oxidative stress disrupts the conversion of proline to HYP by directly damaging or depleting prolyl hydroxylase. This inhibition leads to proline accumulation and reduced HYP synthesis, ultimately contributing to collagen degradation and loss of skin structural integrity [77].

Our results are consistent with this mechanism, as we observed a highly significant increase in proline levels in mouse skin following UV irradiation (Figure 6e). Arginine, a key constituent of the Natural Moisturizing Factor (NMF), plays an important role in enhancing skin hydration and reducing transepidermal water loss (TEWL), thereby reinforcing the skin’s barrier function [78]. In addition, Arginine exerts proliferative and anti-apoptotic effects on skin fibroblasts, supporting wound repair and maintaining dermal structure and resilience [79]. In this study, Arginine levels in mouse skin rose significantly after UV irradiation and exhibited a strong negative correlation with CAT activity (Figure 6f and Figure 7b). This observation aligns with prior reports showing elevated arginine levels in UV-exposed human skin and a marked decrease in CAT activity in a hyperargininemia rat model following arginine supplementation [80,81]. Phosphatidyl choline (PC), a principal component of the phospholipid bilayer, is essential for preserving the integrity and fluidity of skin cell membranes. Choline serves as the fundamental precursor for PC synthesis [82]. Research indicates that UV exposure increases cross-linking of choline metabolites with proteins within cells [83], which is consistent with our findings. We observed that UV irradiation markedly increased choline levels in mouse skin, whereas both prophylactic and therapeutic topical administration of QLBSO effectively reversed this elevation (Figure 6g). This is closely related to unsaturated fatty acids and VE. QLBSO is rich in unsaturated fatty acids (linoleic acid and oleic acid), polyphenols, and vitamin E derivatives that directly neutralize ROS, interrupt the oxidative chain reaction, and protect cell membranes from lipid peroxidation. Inhibiting ROS production reduces the skin’s demand for amino acids such as cysteine and arginine, allowing their levels to return to normal [84].

In summary, QLBSO mitigates oxidative stress and inflammatory responses in mouse skin by modulating specific microbial populations on the skin surface and regulating amino acid metabolism. Through these coordinated effects, QLBSO promotes collagen synthesis, alleviates UV-induced photodamage, and accelerates skin repair. The primary limitation of this study lies in its scope: although we identified key microbial species and amino acids responsive to QLBSO in the prevention and treatment of UV-induced skin photodamage and established correlations among major skin bacteria, amino acids, oxidative stress markers, and inflammatory mediators using Pearson correlation analysis, the overall mechanism remains complex. Skin health depends on multifaceted interactions among the skin microbiota, resident cells, and a wide array of metabolic products, all of which dynamically influence one another. Thus, this study provides only a preliminary mechanistic explanation of QLBSO’s protective and reparative effects against UV-induced photodamage from the perspective of microbiome–metabolite interactions. Future studies should explore in greater depth the intricate relationships among key microbial taxa, amino acid metabolites, and skin cellular processes involved in QLBSO-mediated photoprotection.

## 4. Materials and Methods

### 4.1. Gas Chromatography-Mass Spectrometry (GC/MS) Analysis of QLBSO

A total of 20 μL of QLBSO was dissolved in 0.5 mL of n-hexane. A gas chromatograph (TRACE 1310, Thermo Fisher Scientific Inc., Waltham, MA, USA) coupled with a mass spectrometer (TSQ 8000 EVO, Thermo Fisher Scientific Inc., Waltham, MA, USA) was utilized with a VF50 column (30 m long, 0.32 mm inner diameter, 0.25 μm film thickness). Analysis conditions were as follows: H_2_ gas was used as the carrier gas at a flow rate of 1 mL/min with a split ratio of 1:50. The column temperature program was set as follows: initial temperature of 60 °C held for 1 min, followed by a ramp of 10 °C/min to 180 °C. The temperature was increased at 4 °C/min to 280 °C and held for 20 min. The temperature was raised at 20 °C/min to 300 °C and held for 2 min. The mass spectrometry detection range was 30–600 Da.

### 4.2. Materials and Animal Husbandry

BALB/c mice (8 weeks old, female, license number SCXK(Jing)2019-0008) were obtained from Beijing Huafukang Biological Technology Co., Ltd. (Beijing, China). All animal experiments were conducted in accordance with international ethical standards. The research protocol was reviewed and approved by the Qinghai University Science and Technology Ethics Committee (approval number: SL202501-70). Mice were housed in an SPF-grade animal facility maintained at 23–24 °C with 50 ± 15% relative humidity under a 12 h light/dark cycle. Seed oil of *Lycium barbarum* L. cultivated in Qaidam basin (designated QLBSO, KPGQZY-23-05) was provided by Qinghai Kangpu Biotechnology Co., Ltd. (Xining, China). Vitamin E (designated VE, s13017) is provided by Shanghai Yuanye (Shanghai, China).

### 4.3. Acute Toxicity Study of QLBSO in BALB/c Mice

Following an adaptation period, 40 BALB/c mice (8 weeks old, equal numbers of males and females, 16–20 g) were randomly separated into four groups of 10 mice each: a local intervention blank control group, a QLBSO local intervention group, an oral intervention blank control group, and a QLBSO oral intervention group. Mice were fasted for 12 h before the experiment with access to water. Hair on the dorsal region was removed. The “maximum tolerated dose test” was conducted. For the topical intervention group, a single uniform application of 0.4 mL of QLBSO was applied to the dorsal skin. The oral intervention group received a single gavage dose of 20 mL/kg. The topical and oral intervention blank control groups received equal volumes of saline via the corresponding routes. This was repeated once every 8 h, 3 times a day, for 7 consecutive days, and behavioral, body weight, and skin condition changes in mice were continuously monitored over 7 days [85]. Mice were anesthetized with isoflurane (3%) [86] and euthanized by cervical dislocation. Dissect the mice and carefully examine for any obvious pathological changes in the heart, liver, spleen, and kidneys.

### 4.4. Animal Grouping and Model Establishment

**Figure 8:**
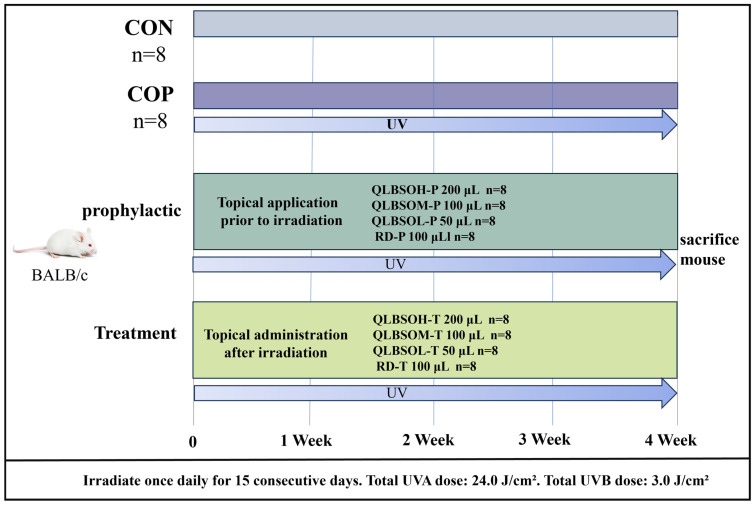
Experimental Design for Photodamaging in BALB/c Mouse Skin.

We investigated the photoprotective effects of QLBSO in a UV-induced BALB/c mouse skin photodamage model using two approaches: Prophylactic topical administration (Prophylactic) and treatment topical administration (treatment). BALB/c mice were randomly assigned to ten groups, with eight mice per group. The formal experiment employed three doses of QLBSO: high (QLBSOH, 200 μL), medium (QLBSOM, 100 μL), and low (QLBSOL, 50 μL). A 100 μL VE solution aliquot served as the reference drug group (RD). Specific groupings and intervention methods are outlined in Table 1 and Figure 8. To prepare the mice for UV exposure, hair was shaved from the mid dorsal region to create an exposed area of approximately 2 × 3 cm^2^, with regular hair removal throughout the study. The irradiation protocol followed methods from the literature with minor adjustments [26]. All mice, except those in the control group (CON), were exposed to mixed UVA and UVB radiation every other day for 15 min sessions, for a total of 15 sessions (UV Accelerated Weathering Test Chamber, Guangdong Aipei Test Equipment Co., Ltd., Dongguan, Guangdong, China). The UVA dose per session was 1.6 J/cm^2^, with a cumulative dose of 24.0 J/cm^2^, while the UVB dose per session was 0.2 J/cm^2^, with a cumulative dose of 3.0 J/cm^2^. For both prophylactic and therapeutic interventions, QLBSO and VE were locally applied at varying volumes 30 min before UV exposure (for prevention) and immediately following UV exposure (for treatment).

### 4.5. Animal Handling and Sample Collection

After the final UV irradiation, we collected skin surface microbiota samples by gently swabbing the dorsal skin of the mice with cotton swabs. The swabs were immediately placed into cryogenic tubes and stored at −80 °C for subsequent microbiota analysis. Mice were fasted for 12 h, with free access to water, and then anesthetized with isoflurane (3%) [86]. Following anesthesia, they were euthanized by cervical dislocation. We quickly harvested dorsal skin tissue, removed any adherent adipose tissue, and thoroughly rinsed the samples with phosphate-buffered saline (PBS, Qinghai Rhein Bio Technology Co., Ltd., China) to remove residual blood. Dorsal skin samples (approximately 1 × 1 cm^2^) were fixed in 4% paraformaldehyde for histopathological analysis. The remaining skin tissue was stored at −80 °C for future studies.

### 4.6. Histopathological Evaluation of Skin Tissue

After tissue dehydration, embedding, sectioning, and washing, we stained the mouse skin tissue with hematoxylin and eosin (H&E). A microscopic imaging system was used to assess skin tissue thickness and keratinization at 100× magnification. Inflammatory cell infiltration and hemorrhagic symptoms were examined at 400× magnification. Using a glass slide scanning imaging system (SQS-600P, Shenzhen Shengqiang Technology Co., Ltd., Shenzhen, China), the epidermal thickness and dermal thickness of the skin were measured in parallel 10 times, followed by intergroup statistical analysis. Additionally, we performed Masson’s trichrome staining using alizarin red and aniline blue to assess fibrous tissue proliferation in the skin, which was then observed under a microscope at 400× magnification.

### 4.7. Determination of Oxidative Stress Markers, Inflammatory Markers, and Matrix Metalloproteinases in Skin Tissue

We prepared skin tissue homogenates by adding nine volumes of PBS to mouse skin samples and homogenizing to yield a 10% (*w*/*v*) tissue suspension using a cryogenic homogenizer (CBCL-24; Shanghai Cebo Biotechnology Development Centre, Shanghai, China). We centrifuged the homogenates at 10,000 rpm for 10 min at 4 °C and collected the supernatant for analysis. We measured SOD and CAT activities using a microplate visible-light assay (Nanjing Jiancheng Biotechnology Institute, Nanjing, China) [33]. We quantified interleukin-1β (IL-1β), interleukin-6 (IL-6), and tumor necrosis factor-α (TNF-α) using ELISA kits (Proteintech Group, Inc., Wuhan, China), and measured Matrix Metalloproteinase-1 (MMP-1) and Matrix Metalloproteinase-3 (MMP-3) by ELISA (Elabscience Biotechnology Co., Ltd., Wuhan, China) [40].

### 4.8. Skin Microbiome Analysis

We analyzed microbial differences on the skin surfaces of four mouse groups (CON, COP, QLBSOH-P, and QLBSOH-T) using 16S rDNA high-throughput sequencing. The procedures were as follows. We extracted whole-genome DNA from mouse skin microbiota samples and quantified the DNA concentration using a NanoDrop (Thermo Fisher Scientific, Waltham, MA, USA) spectrophotometer. We assessed DNA integrity by agarose gel electrophoresis. To characterize microbial composition and diversity, we amplified target sequences corresponding to microbial ribosomal RNA or specific gene fragments. Primers were designed from conserved regions of these sequences and incorporated sample-specific barcode identifiers. PCR amplification targeted variable regions—single or consecutive—of rRNA genes or other taxonomically informative gene fragments. We quantified PCR products fluorometrically using the Quant-iT PicoGreen dsDNA Assay Kit and a microplate reader (BioTek, FLx800, Winooski, VT, USA). Based on the fluorescence quantification results, we pooled samples in appropriate ratios to meet sequencing depth requirements. We constructed sequencing libraries using Illumina’s TruSeq Nano DNA LT Library Prep Kit (San Diego, CA, USA). For raw sequences with sequencing library concentrations ≥ 2 nM, library and sample classification were performed according to index and barcode information [87]. We trimmed barcode sequences and processed reads by denoising clustering using the QIIME2 dada2 (2019.4) workflow. We visualized microbial composition at multiple taxonomic levels to describe the overall community structure. At the ASV level, we calculated distance matrices for each sample. We applied several unsupervised ordination and clustering methods, along with corresponding statistical tests, to evaluate β-diversity differences and their significance across groups. At the species composition level, we applied both unsupervised and supervised ranking, clustering, and modeling approaches, integrated with statistical analyses, to assess differences in species abundance among groups. Finally, we identified microbial biomarkers using the LEfSe algorithm.

### 4.9. Non-Targeted Metabolomics Analysis

We conducted non-targeted metabolomics to identify metabolites in skin tissue from four mouse groups: CON, COP, QLBSOH-P, and QLBSOH-T. The procedure was as follows. After slowly thawing the samples at 4 °C, we added each to prechilled methanol/acetonitrile/water (2:2:1, *v*/*v*/*v*). Samples were vortexed, sonicated at low temperature for 30 min, and incubated at −20 °C for 10 min. We centrifuged at 14,000 rpm for 20 min at 4 °C, collected the supernatant, and vacuum-dried it. For mass spectrometry, we resuspended the dried extract in 100 μL of acetonitrile/water (1:1, *v*/*v*), vortexed, centrifuged at 14,000 rpm for 15 min at 4 °C, and injected the supernatant for analysis. Chromatographic separation was performed on a Vanquish LC ultra-high-performance liquid chromatography (UHPLC Agilent/Thermo Scientific, Waltham, MA, USA) system equipped with a HILIC column. The column temperature was maintained at 25 °C, with a flow rate of 0.5 mL/min and an injection volume of 2 μL. The mobile phase consisted of (A) water containing 25 mM ammonium acetate and 25 mM ammonium hydroxide and (B) acetonitrile. Mass spectrometry was carried out using an Orbitrap Exploris™ 480 instrument (AB SCIEX/Thermo Scientific, Waltham, MA, USA) operated in both positive and negative ion modes. We used XCMS software (v4.2.3) for peak alignment, retention time correction, and peak area extraction. Processed data underwent metabolite identification and preprocessing. Metabolite structures were confirmed by matching retention times, molecular weights (mass errors ≤ 10 ppm), fragmentation patterns, and collision energies. Multivariate statistical analyses, including Principal Component Analysis (PCA) and Partial Least Squares Discriminant Analysis (PLS-DA), were applied to identify differentially expressed metabolites.

### 4.10. Amino Acid-Targeted Metabolomics Analysis

We performed targeted metabolomics to quantify amino acid alterations in skin tissues from four mouse groups: CON, COP, QLBSOH-P, and QLBSOH-T. Differentially expressed amino acids were screened as follows. After thawing the skin tissue samples (as described in Section 4.9), we mixed an appropriate amount of each sample with a solution containing isotope-labeled internal standards and pre-chilled methanol/acetonitrile/water (2:2:1, *v*/*v*/*v*). The mixture was vortexed and incubated at −20 °C for 1 h to precipitate proteins. We then centrifuged the samples at 14,000 rpm for 20 min at 4 °C and collected the supernatant, which was vacuum-dried. For mass spectrometry, we resuspended the dried extract in 100 μL of acetonitrile/water (1:1, *v*/*v*), vortexed, centrifuged at 14,000 rpm for 15 min at 4 °C, and injected the supernatant for analysis. We prepared a mixed standard stock solution from multiple amino acid standards and diluted it in serial steps to generate a calibration curve with a concentration gradient. Each gradient solution was processed in parallel using the same sample preparation protocol. Mass spectrometric analysis was performed on a 6500+ QTRAP system (SCIEX) operated in positive ion mode. We used Multiquant 3.0.2 software to extract chromatographic peak areas and retention times. Retention times were corrected and verified using standard reference compounds corresponding to the target metabolites. We identified differentially expressed amino acids through cluster analysis and one-way analysis of variance (ANOVA).

### 4.11. Statistical Analysis

We analyzed all experimental data using GraphPad Prism 9.5.1 software. ANOVA was applied to assess statistical differences, and results are expressed as mean ± standard deviation (SD). We considered differences statistically significant at *p* ≤ 0.05 or *p* ≤ 0.01. Bioinformatics analyses were performed using the OmicStudio platform on the Lianchuan Bio Cloud Platform (https://www.omicstudio.cn/tool, accessed on 22 June 2025).

## 5. Conclusions

Both prophylactic and therapeutic topical administration of QLBSO could improve UV-induced photodamage in mouse skin by enhancing antioxidant enzyme activity and reducing levels of inflammatory factors and matrix metalloproteinases in skin tissue. Furthermore, QLBSO alleviates oxidative stress and inflammatory responses in the skin by modulating the skin microbiota, including *Kineothrix*, and amino acid metabolites of cysteine, cystine, glycine, and choline. This study not only provides new directions for the utilization of QLBSO and expands its application scope, but also offers novel insights into QLBSO’s role in protecting against and treating UV-induced photodamage. This work lays a theoretical foundation for subsequent related research.

## Figures and Tables

**Table 1:** Mouse Groups and Intervention Methods.

Topical Administration Time	Grouping	Processing	Topical Dose	Dose/μL
/	Control (CON)	/	/	0
/	Control positive (COP)	UV	/	0
Prophylactic (30 min before irradiation)	QLBSO High-Dose prophylactic Group (QLBSOH-P)	UV	QLBSO	200
	QLBSO Medium-Dose prophylactic Group (QLBSOM-P)	UV	QLBSO	100
	QLBSO Low-Dose prophylactic Group (QLBSOL-P)	UV	QLBSO	50
	Prophylactic Reference Drug Group (RD-P)	UV	VE	100
Treatment (after irradiation)	Treatment Reference Drug Group (RD-T)	UV	VE	100
	QLBSO High-Dose therapeutic Group (QLBSOH-T)	UV	QLBSO	200
	QLBSO Medium-Dose therapeutic Group (QLBSOM-T)	UV	QLBSO	100
	QLBSO Low-Dose therapeutic Group (QLBSOL-T)	UV	QLBSO	50

## Data Availability

All data supporting the reported results are contained within the article. For specific data, please refer to Table S1. The 16S rDNA gene high-throughput sequencing data have been uploaded to the NCBI database (PRJNA1369097).

## Supplementary Materials

The following supporting information can be downloaded at: https://www.mdpi.com/article/10.3390/ijms27020731/s1.
