# Supplementary material for: Seed Oil of Lycium barbarum L. from Qaidam Basin Prevents and Treats UV-Induced Photodamage in BABL/c Mice Skin by Modulating Skin Microbiome and Amino Acid Metabolism
Source: Int J Mol Sci. 2026 Jan 11;27(2):731. doi: 10.3390/ijms27020731 (PMC12841451; doi:10.3390/ijms27020731)
Supplement: Supplementary file 1 [file ijms-27-00731-s001.zip › ijms-4063033-supplementary.pdf]

Supplementary Data

For

Seed Oil of *Lycium barbarum* L. from Qaidam basin Prevents and Treats UV-Induced Photodamage in BABL/c Mice Skin by Modulating Skin Microbiome and Amino Acid Metabolism

Le Han[1], Yongjing Yang [2,*],Benyin Zhang[1], Yuting Wang1, Yiming Ji[1], Shasha Du[1], Yongqiang Zou[3].

1 College of Eco-Environmental Engineering, Qinghai University, Xining 810016, China;

YS230713000141@qhu.edu.cn (L.H.)

2 College of Pharmacy, Qinghai University, Xining 810016, China

3 Qinghai Institute for Endemic Disease Prevention and Control, 810016, China

* Correspondence: 2016990031@qhu.edu.cn

Table S1. Original figure data for the article

Epidermal thickness (μm)									
**CON**	**COP**	**QLBSOH-P**	**QLBSOM-P**	**QLBSOL-P**	**RD-P**	**QLBSOH-T**	**QLBSOM-T**	**QLBSOL-T**	**RD-T**
12.89	215	66.31	82.56	87.65	77.42	40.02	42.5	85.72	59.19
12.89	217.5	65.92	70.86	74.77	68.3	53.49	41.42	91.13	48.2
15.23	203.42	82.2	91.5	66.95	51.09	44.1	48.95	93.84	33.82
19.92	210.04	74.89	87.38	80.2	59.15	46.55	43.57	83.91	32.89
14.65	242.21	71.24	83.25	80.2	59.69	57.68	60.01	79.31	30.62
19.33	246.94	80.98	83.49	70.64	55.93	58.21	46.62	75.97	31.45
13.48	314.12	89.5	83.41	66.53	76.04	57.16	66.44	56.41	30.62
9.72	240.32	48.71	100.42	86.42	70.57	45.55	55.63	57.31	31.87

Dermal thickness (μm)									
**CON**	**COP**	**QLBSOH-P**	**QLBSOM-P**	**QLBSOL-P**	**RD-P**	**QLBSOH-T**	**QLBSOM-T**	**QLBSOL-T**	**RD-T**
230	505	300.59	252.06	222.37	304.37	217.06	271.74	238.24	311.01
215	587.5	287.26	272.16	239.66	314.25	175.9	241.78	287.14	256.46
242.24	422.5	266.65	337.11	238.43	274	217.06	231.26	304.66	306.06
178.5	447.5	292.11	278.35	226.07	293.77	218.18	275.77	297.86	308.54
233	472.5	282.41	310.82	208.78	296.6	222.13	226.62	326.2	304.4
219.4	477.5	303.02	281.44	224.84	300.13	224.95	235.68	308.31	262.48
240.22	432.5	293.96	284.54	217.43	358.88	252.16	238.7	276.8	261.19
201.41	497.5	315.64	306.18	221.13	323.2	236.4	221.43	234.22	362.31

SOD (U/mgprot)									
**CON**	**COP**	**QLBSOH-P**	**QLBSOM-P**	**QLBSOL-P**	**RD-P**	**QLBSOH-T**	**QLBSOM-T**	**QLBSOL-T**	**RD-T**
267.2049	106.3006	175.6767	214.3723	193.224	210.4098	247.7433	186.3159	186.1803	182.3725
234.0285	157.2051	164.7877	173.0912	197.3062	202.1763	252.6491	156.6018	195.2422	237.3899
272.5849	104.8034	171.3211	178.885	176.8952	225.9618	246.1081	238.5165	179.5899	234.3334
225.9585	172.9256	217.7811	225.96	189.8221	222.3025	249.3786	197.5592	223.2516	240.4464
234.9251	110.7922	182.2102	220.8904	199.3473	193.0281	183.9678	236.9104	199.3612	236.3711
243.8918	174.4228	187.2917	211.4754	175.5344	284.5106	187.2384	187.119	173.8232	243.503
172.1589	111.5408	214.1514	218.7177	209.5527	212.2394	182.3325	166.2388	195.2422	241.4653

270.7916	172.177	208.3439	161.5035	168.7308	201.2615	260.0078	252.9721	228.1945	250.6349

CAT (U/mgprot)									
CON	COP	QLBSOH-P	QLBSOM-P	QLBSOL-P	RD-P	QLBSOH-T	QLBSOM-T	QLBSOL-T	RD-T
41.89168	16.48652	18.53624	36.14739	22.06865	24.34482	23.48738	24.89578	21.03526	23.65767
41.17619	8.422463	16.79846	21.03541	13.05661	23.17685	24.69437	26.46578	20.57512	30.64929
42.24942	11.43902	14.88691	25.31184	18.94701	22.62937	22.34563	30.21457	27.60878	26.78764
36.59709	8.213395	15.35032	19.87962	18.62128	23.72433	27.56504	24.54333	20.70659	25.81206
33.6636	11.08062	16.74054	20.89094	17.58978	23.06735	22.05204	24.287	21.82409	39.10426
46.47079	19.20441	24.4157	21.29547	13.49092	28.6517	25.02058	27.49109	17.61703	23.73897
44.61052	15.88919	18.85483	21.69999	18.10553	23.83383	25.02058	20.69842	24.48636	28.45424
52.33777	15.29185	13.67047	22.16231	19.97851	22.84836	19.89903	25.79292	30.89554	28.21035

IL-1β (pg/mL)									
CON	COP	QLBSOH-P	QLBSOM-P	QLBSOL-P	RD-P	QLBSOH-T	QLBSOM-T	QLBSOL-T	RD-T
1127.38418	6788.15589	1766.4028	1147.17911	4887.4036	1312.8953	1353.73275	1951.31308	3989.43466	1203.60314
513.43386	7154.59713	2135.95262	1706.54658	1823.15126	1748.20471	563.13433	1775.52148	3004.47844	706.67935
596.4628	3693.08337	1882.26756	604.0597	2379.98801	1706.54658	1289.19501	3495.95007	4198.48354	1427.56814
760.91512	8565.86305	2796.09439	982.46474	1688.51956	1902.71647	1282.43982	1908.30487	1009.89264	2476.97892
799.9288	4133.30761	1579.65467	1441.40092	3070.95287	5816.34191	686.64626	2276.52509	3017.72079	1198.60426
1268.9511	5402.22929	3479.3789	934.34708	1850.79352	1450.06184	610.14321	2339.99869	1324.77892	1693.92227
1488.31196	3086.55859	2534.23089	963.17436	1786.48125	623.85075	1453.52955	1744.57136	1178.64815	1158.75478
1519.78107	8051.70561	3823.53227	3911.0254	1824.99034	2669.09949	1268.9511	3517.31453	4982.94456	2392.03303

TNF-α (pg/mL)									
CON	COP	QLBSOH-P	QLBSOM-P	QLBSOL-P	RD-P	QLBSOH-T	QLBSOM-T	QLBSOL-T	RD-T

102.50054	905.66829	394.13472	360.16838	518.00981	181.13881	326.7173	265.86097	493.12946	149.95801
71.70855	2433.12852	313.84924	276.62445	632.44783	179.43652	358.29641	124.36429	463.59294	226.04195
97.69982	1011.39549	237.45584	166.72997	606.48142	165.8867	217.31236	163.35983	436.341	117.843
60.91373	2032.17829	318.43587	355.49144	793.39138	144.96539	394.13472	168.41794	447.01073	193.10709
114.59575	1488.98776	338.73699	175.18908	541.11357	162.51851	334.10599	127.63812	400.79862	117.843
92.92123	976.16065	126.81885	151.62626	593.07494	160.83735	262.28656	126.00012	496.10231	171.79968
109.74195	1208.2831	391.28466	190.53481	575.64241	217.31236	333.181	213.83291	397.9403	168.41794
115.40671	850.14753	256.04759	369.55206	567.47766	354.55724	253.3801	154.13241	401.75218	256.9376

IL-6 (pg/mL)									
**CON**	**COP**	**QLBSOH-P**	**QLBSOM-P**	**QLBSOL-P**	**RD-P**	**QLBSOH-T**	**QLBSOM-T**	**QLBSOL-T**	**RD-T**
734.71693	5970.01036	3397.4371	4351.60578	4125.12298	3131.47181	1428.96367	1480.01845	1198.21918	1378.4186
766.36498	4749.20019	3041.7102	3508.29362	5341.81357	3083.67416	888.45808	1597.30574	2340.65669	618.64479
302.86935	5754.98012	3322.31762	2941.80189	3763.86121	2330.42396	597.43858	953.18074	770.11025	613.32636
359.52621	5352.15917	2875.82823	4285.07283	4304.04224	3417.76286	994.97764	541.78311	785.13723	2173.64264
331.68692	6118.88303	2864.88179	4181.31249	4576.21994	4103.34901	1437.80626	367.34967	2000.77863	707.07244
1133.49552	5980.85005	4576.21994	4585.94406	2686.29636	2664.91017	1585.91689	880.6935	1792.13323	977.00717
361.0878	5776.33234	3276.38485	4726.20927	3818.17519	2619.65184	1435.59417	705.23891	911.85686	663.39714
277.59994	6361.85686	3587.83017	2761.59191	4858.17972	4348.42865	1156.35052	604.48727	2848.48849	1518.07992

MMP-1 (pg/mL)									
**CON**	**COP**	**QLBSOH-P**	**QLBSOM-P**	**QLBSOL-P**	**RD-P**	**QLBSOH-T**	**QLBSOM-T**	**QLBSOL-T**	**RD-T**
1922.531862	17964.64747	8529.406468	10484.05355	14502.95943	4604.480662	4729.663382	13040.17723	12269.38652	3995.64179
1550.312522	18208.23896	9783.451468	10192.59415	15615.0016	3643.845816	4616.353428	11489.51294	14262.66854	2096.248454
653.54705	17563.57425	6517.853754	10391.99823	15186.82415	3058.826172	6403.528572	10027.92472	13799.08035	4063.793396
1693.447052	17540.83932	9638.21129	11782.01739	14929.37788	6150.903736	8643.494032	9726.84118	16212.05748	2341.45347
1171.201272	17001.06411	7750.25156	11604.26455	16592.55259	2239.683264	7440.79508	8804.405746	15965.10765	2774.954698
834.397444	16956.5861	7706.74045	13614.25724	15794.7433	6423.644554	5026.238012	9240.370198	13163.34052	2244.50402
1497.349394	17906.97709	7852.198086	12114.7746	15858.49352	3782.243822	4819.78334	10003.34117	12333.36867	2608.277592
2100.992478	17746.16554	6955.60589	11997.27687	15773.52949	2283.161744	6276.712768	9807.763038	13517.5723	3632.842206

MMP-3 (ng/mL)

CON	COP	QLBSOH-P	QLBSOM-P	QLBSOL-P	RD-P	QLBSOH-T	QLBSOM-T	QLBSOL-T	RD-T
2.457893	20.311418	5.041509	11.266751	17.122307	2.8315	6.021475	4.315542	6.739102	3.266057
1.346342	42.676388	5.865529	8.422126	20.712129	3.019596	4.669102	4.127735	7.716797	2.699014
2.146212	23.904334	6.004051	12.525987	13.417311	2.310534	4.376278	3.979621	7.716797	4.945322
2.613714	19.854357	6.126537	8.275966	16.611985	2.725389	5.484071	4.023859	6.384144	3.724854
1.369792	20.983996	5.13036	12.345452	13.716017	3.23841	3.818833	4.794584	7.568319	6.004051
1.721218	24.932298	6.465177	12.017682	13.791581	2.952064	3.667396	4.467971	8.275966	2.858183
1.369792	30.982574	7.276332	8.275966	12.864967	2.685849	5.856923	4.285291	7.047386	2.731992
1.120075	29.885771	6.073896	10.118489	16.274088	2.190221	3.489653	6.170576	7.617625	3.47555

Firmicutes_A			
CON	COP	QLBSOH-P	QLBSOH-T
0.204304	0.421363	0.245721	0.091317
0.08331	0.188138	0.32383	0.022705
0.183973	0.210778	0.263786	0.028187
0.008525	0.414154	0.352859	0.016576
0.009086	0.462111	0.317614	0.027087

Kineothrix			
CON	COP	QLBSOH-P	QLBSOH-T
0.022705	0.050698	0.033648	0.001727
0	0.018648	0.013727	0.001101
0.015345	0.03177	0.017439	0.008741
0	0.068936	0.059331	0.000496
0.000324	0.045928	0.043425	0.003518

Cysteine			
CON	COP	QLBSOH-P	QLBSOH-T
0.27034609	14.4328325	8.39084008	0.40091045
0.28444944	15.0156704	8.66758378	0.38867647
0.2831654	14.7110435	8.77938595	0.38291326
0.28129331	15.1157425	9.37322919	0.41276784

Cystine			
CON	COP	QLBSOH-P	QLBSOH-T
13.4243276	281.306644	161.271027	1.6542143
13.3432736	303.498716	173.005266	1.42680873
13.3143088	277.596386	165.052281	1.41823854
13.7026339	301.205405	175.85492	1.30569094

Glycine			
**CON**	**COP**	**QLBSOH-P**	**QLBSOH-T**
3054.81575	5713.6081	4856.4927	3878.03344
3204.39964	5720.74803	4813.97878	3672.43569
3146.93604	5812.45904	4962.61306	3921.91336
3219.00718	5931.28139	4880.81665	3625.77103

Proline			
**CON**	**COP**	**QLBSOH-P**	**QLBSOH-T**
292.58415	401.019633	383.08501	291.822725
290.753095	400.82484	383.423441	281.601081
294.514853	393.904061	383.367042	295.314598
300.551043	410.044159	386.736549	281.869282

Arginine			
**CON**	**COP**	**QLBSOH-P**	**QLBSOH-T**
1562.73472	2925.19984	2484.49991	2241.39708
1629.87861	2948.29662	2471.79726	2198.40507
1582.64351	3022.63534	2590.79266	2333.24958
1622.44035	2911.38538	2557.09149	2200.71344

Choline			
**CON**	**COP**	**QLBSOH-P**	**QLBSOH-T**
59198.0602	68085.7019	58469.0626	41482.4561
60092.3676	66937.5216	57138.5306	40179.097
59789.2273	65445.0775	58863.6264	41870.239
60474.6518	69217.3171	57097.625	42022.3633

Table S2: Abbreviation Reference Table

Abbreviation	Full name	Abbreviation	Full name
QLBSO	Seed Oil Of *Lycium Barbarum* L. From Qaidam Basin	ROS	Reactive Oxygen Species
UV	Ultraviolet	CAT	Catalase

SOD	Superoxide Dismutase	TNF-Α	Tumor Necrosis Factor-Α
Mmps	Matrix Metalloproteinases	IL-6	Interleukin-6
IL-1Β	Interleukin-1Β	MMP-3	Matrix Metalloproteinases-3
MMP-1	Matrix Metalloproteinases-1	LEfSe	LDA Effect Size
GC/MS	Gas Chromatography-Mass Spectrometry	PLS-DA	Partial Least Squares–Discriminant Analysis
PCA	Principal Component Analysis	ICAM-1	Intercellular Adhesion Molecule-1
MHC-I	Major Histocompatibility Complex Class I	PC	Phosphatidyl Choline
VCAM-1	Vascular Cell Adhesion Molecule 1	ECM	Extracellular Matrix
COX	Cyclooxygenase	5-LOX	5-Lipoxygenase
H_2S	Hydrogen Sulfide	LPS	Lipopolysaccharides
GSH	Glutathione	HYP	Hydroxyproline
NMF	Natural Moisturizing Factor	TEWL	Transepidermal Water Loss
CON	Control Group	RD-P	Reference Drug Prophylactic Group
COP	Control Positive Group	RD-T	Reference Drug Therapeutic Group
QLBSOH-P	QLBSO High-Dose prophylactic Group	QLBSOH-T	QLBSO High-Dose therapeutic Group
QLBSOM-P	QLBSO Medium-Dose prophylactic Group	QLBSOM-T	QLBSO Medium-Dose therapeutic Group
QLBSOL-P	QLBSO Low-Dose prophylactic Group	QLBSOL-T	QLBSO Low-Dose therapeutic Group
